# Hotspots for rockfishes, structural corals, and large-bodied sponges along the central coast of Pacific Canada

**DOI:** 10.1038/s41598-021-00791-9

**Published:** 2021-11-09

**Authors:** Alejandro Frid, Madeleine McGreer, Kyle L. Wilson, Cherisse Du Preez, Tristan Blaine, Tammy Norgard

**Affiliations:** 1 Central Coast Indigenous Resource Alliance, Campbell River, BC Canada; 2 School of Environmental Studies, University of Victoria, Victoria, BC Canada; 3 Institute of Ocean Sciences, Fisheries and Oceans Canada, Sidney, BC Canada; 4 Pacific Biological Station, Fisheries and Oceans Canada, Nanaimo, BC Canada

**Keywords:** Biodiversity, Conservation biology, Marine biology

## Abstract

Biological hotspots are places with outstanding biodiversity features, and their delineation is essential to the design of marine protected areas (MPAs). For the Central Coast of Canada’s Northern Shelf Bioregion, where an MPA network is being developed, we identified hotspots for structural corals and large-bodied sponges, which are foundation species vulnerable to bottom contact fisheries, and for Sebastidae, a fish family which includes species that are long-lived (> 100 years), overexploited, evolutionary distinctive, and at high trophic levels. Using 11 years of survey data that spanned from inland fjords to oceanic waters, we derived hotspot indices that accounted for species characteristics and abundances and examined hotspot distribution across depths and oceanographic subregions. The results highlight previously undocumented hotspot distributions, thereby informing the placement of MPAs for which high levels of protection are warranted. Given the vulnerability of the taxa that we examined to cumulative fishery impacts, prospective MPAs derived from our data should be considered for interim protection measures during the protracted period between final network design and the enactment of MPA legislations. These recommendations reflect our scientific data, which are only one way of understanding the seascape. Our surveys did not cover many locations known to Indigenous peoples as biologically important. Consequently, Indigenous knowledge should also contribute substantially to the design of the MPA network.

## Introduction

Biodiversity loss affects all human societies^1^, yet its harm can be disproportionately greater for Indigenous peoples who derive food security and cultural identity from local ecosystems^2,3^. In the latter part of the twentieth century, First Nations along the Central Coast of British Columbia, Canada, began to experience rapid declines in the abundance of marine species inherent to traditional foods, including Pacific salmon (*Oncorhynchus* spp*.*)^4^, eulachon (*Thaleichthys pacificus*)^5^, and yelloweye rockfish (*Sebastes ruberrimus*)^6^. These species have yet to recover. The rise of commercial and recreational fisheries, combined with anthropogenic climate change^2,5,7^, have influenced these negative trends, amplifying the challenge of cultural revitalization in the aftermath of colonialism^2,8^.

Many species of cultural significance play important ecosystem roles. They include upper-level predators (e.g., yelloweye rockfish^9^) that may indirectly benefit smaller organisms via trophic cascades^10^, anadromous species that transport nutrients from offshore areas to estuaries and riparian ecosystems (e.g., Pacific salmon^11^, eulachon^12^), and foundation species that create biogenic habitats (e.g., kelps^13^). Further, some of these species are evolutionary distinctive and vulnerable to large-scale fisheries (e.g. yelloweye rockfish^14^). Consequently, losses of biological and cultural diversity are inextricably linked.

Evidence from diverse regions of the world indicates that networks of Marine Protected Area (MPAs) can help reverse negative trends and support sustainable ecosystems, economies, and cultures^15^. For example, fish and invertebrates inside MPAs become more abundant and grow to greater size and age than in exploited areas^16^. Consequently, MPAs may increase the productivity of exploited species, promoting resilience to climate change and the export of larvae and adults to fished areas^16,17^. MPAs or spatial fishery closures can also protect foundation species vulnerable to bottom-contact fisheries, such as corals and sponges^18–20^.

MPAs, however, often have been established without involving Indigenous peoples, undermining their governance structures and curtailing their access to traditional harvest areas, thereby hampering cultural diversity. Accordingly, there is growing recognition that Indigenous peoples should lead their own spatial planning processes or, at the very least, be legitimate partners in MPA governance, research, design, and implementation^21,22^.

The effectiveness of MPA networks also depends on the extent to which the location and protection levels of individual MPAs prioritize conservation objectives over extractive activities^23^, and on the monitoring and enforcement of regulations limiting such activities^24^. Consequently, commercial fishers and other stakeholders may lose access to areas they used previously. If convinced of the conservation benefits, stakeholders may accept displacement and support spatial protections; if unconvinced, they may stymie implementation of the MPA network^25^.

The delineation of biological hotspots—places with outstanding biodiversity or ecological features—can help justify spatial protections^26–28^, potentially reducing stakeholder conflicts. Hotspot criteria include (but are not limited to) endemism^27,28^, localized prey aggregations or oceanographic processes that persist over time and support predators^29^, and species assemblages that are ecologically important and vulnerable to extractive activities^18–20,30^.

**Figure 1:**
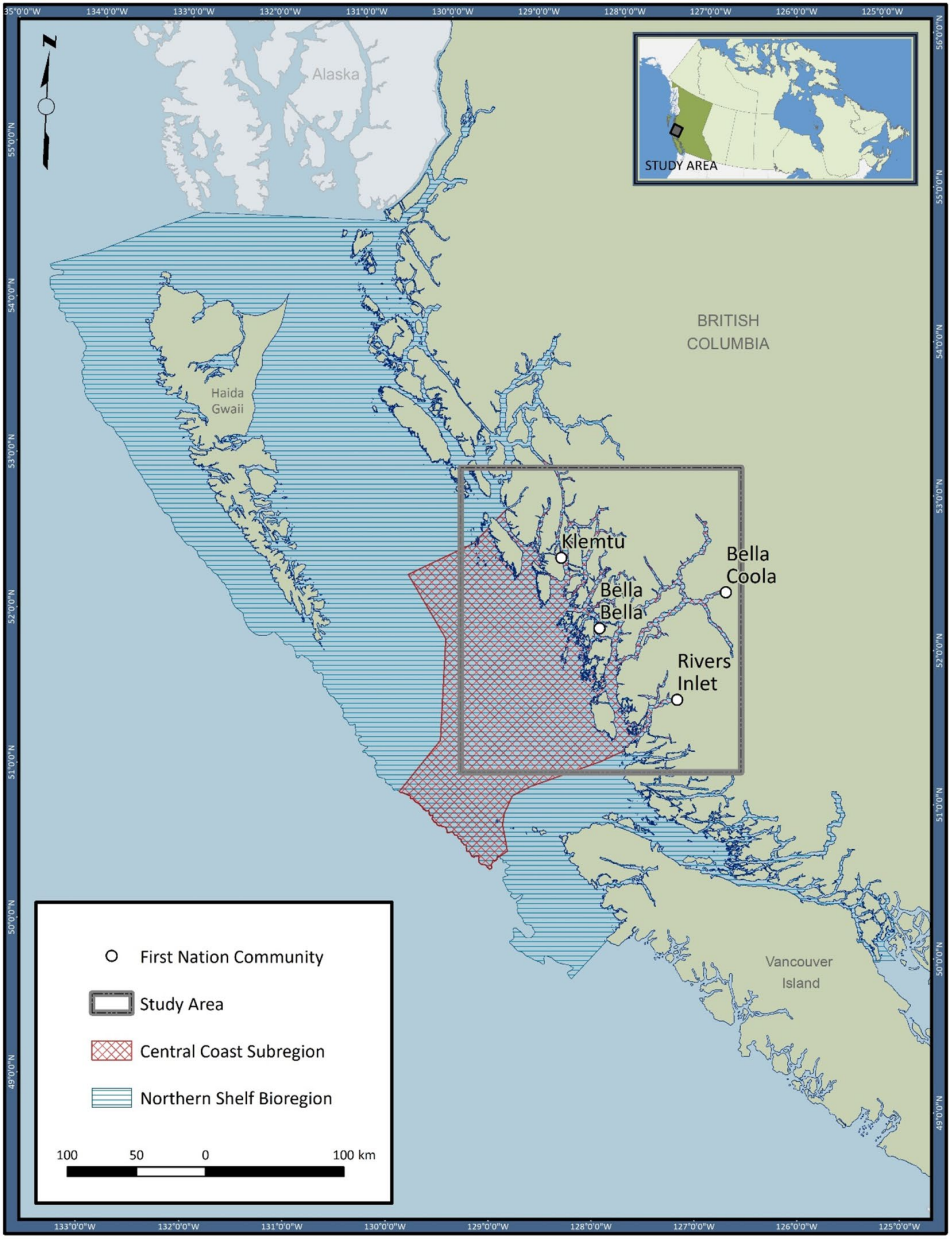
Map of the study area in the context of the Northern Shelf Bioregion, Pacific Canada. (Figure was created with ArcGIS Desktop, Version 10.8.1: https://www.esri.com/en-us/arcgis/products/arcgis-desktop/overview).

The ongoing development of an MPA network for Canada’s Northern Shelf Bioregion^22^ (Fig. 1) is a potential nexus for Indigenous governance and the protection of biological hotspots. The MPA process intends to honour Indigenous rights and title to their traditional territories, such that 17 First Nations and the federal government are governance partners responsible for network design and implementation^22^. The First Nations involved have used their traditional and local knowledge to identify areas of cultural, spiritual, and biological importance to be protected by the MPA network. These Nations also support Western science as a knowledge system complementary to their own^31^.

The Wuikinuxv, Nuxalk, Heiltsuk and Kitasoo/Xai’xais First Nations live along the Central Coast subregion of the Northern Shelf Bioregion (Fig. 1) and are among the governance partners for the MPA network. Collaborating under the umbrella of the Central Coast Indigenous Resource Alliance (CCIRA), since 2013 they have been using fishery-independent methods (dive and towed video transects, hook and line sampling) to survey biodiversity features in their territories^32–34^. The surveys encompass oceanic and inland waters at depths of 5 m to 200 m. Additional research in 2018 included a collaboration with Fisheries and Oceans Canada (DFO: the federal aquatic ecosystem and resource management agency)—which contributed technical capacity and infrastructure (large vessel, crew, and the towed video camera described by Gale et al.^35^) to sample depths of 200 m to 500 m.

The surveys have targeted locations where fish of cultural significance, such as rockfish (*Sebastes* spp.), are expected on the basis of local Indigenous knowledge, yet have also documented foundation species, such as structural corals (i.e., taxa that are erect and branching, including the orders Antipatharia, Alcyonacea, and Anthoathecata) and large-bodied sponges (taxa that are erect and vase- or mound-shaped, including the classes Hexactinellidae and Demospongiae)^32^. The data span three distinct oceanographic areas, known as Upper Ocean Subregions^36^, for which we can identify biological hotspots in support of MPA network planning and implementation. Notably, our extensive surveys include the Mainland Fjords Upper Ocean Subregion, where data gaps curtailed earlier analyses of biodiversity distributions^37^.

Rockfishes are well-suited for hotspot delineation at small spatial scales. They include sedentary, long-lived species (maximum lifespans > 100 years)^38^ that occupy high trophic positions^9^, are large-bodied and evolutionarily distinctive^14^. Some rockfishes have been harvested sustainably by coastal peoples for over 2500 years^39,40^. The genus also includes smaller, shorter-lived species that are planktivorous and important prey to larger predators (e.g., *S*. *emphaeus*; *S*. *jordani*)^38^. Marked declines in the abundance and body sizes of culturally-significant rockfishes began in the 1980s, concurrently with a surge in commercial fishery activity^6,41^. Body size declines appear to be ongoing^42^, likely signaling overexploitation and loss of population productivity^17^.

As sessile foundation species vulnerable to bottom contact fishing gear, structural corals and large-bodied sponges also are suited for hotspot delineation at small spatial scales. In addition to forming biogenic habitats for other species^20,43,44^, corals and sponges influence ecosystems through water filtration, carbon sequestration and basal support for food webs^19,45–47^.

In this study we identify hotspots for the fish family Sebastidae—which includes rockfish and shortspine thornyhead (*Sebastolobus alascanus*)—and for structural corals and large-bodied sponges in the Central Coast of the Northern Shelf Bioregion. We standardized and combined abundance data from different survey types conducted by CCIRA-member Nations and collaborating DFO scientists, and weighted species for conservation prioritization according to proxies for ecological role and vulnerability to fisheries. Rockfishes with available data also were weighted by their evolutionary distinctiveness and depletion level. We then calculated hotspot indices that accounted for the spatial overlap and relative abundance of different species and examined hotspot distributions across Upper Ocean Subregions while accounting for the maximum depths sampled. These analyses support explicit goals of the MPA network^48^ for biodiversity protection (Goal 1, particularly objectives 1.1, 1.2, 1.3 and 1.5), conservation of species exploited by commercial and/or recreational fishers (Goal 2), and conservation of species that are culturally significant to Indigenous peoples (Goal 5) (Appendix S1). Moreover, four of the authors (AF, MM, KLW, and TB) work directly for the four First Nations of the Central Coast, with who they met regularly to ensure that sampling design and study objectives were consistent with the Nations’ priorities for conservation.

## Methods

The Wuikinuxv, Kitasoo/Xai’xais, Heiltsuk and Nuxalk First Nations hold Indigenous rights to their territories, where all data were collected. Scientific staff who are members of these Nations or who work directly for them had direct approvals from Indigenous rights holders and were exempt from other research permit requirements. Collaborating DFO scientists worked in partnership with the First Nations to collect data in their territories..

Sampling targeted rocky reefs, the preferred habitat for most Sebastidae^38^, which we located through local Indigenous knowledge or a bathymetric model^49^. Data were collected by four fishery-independent methods—shallow diver transects, mid-depth video transects, deep video transects, and hook-and-line sampling—detailed in earlier publications^32–35,50,51^ and summarized in Table 1. Data had a spatial resolution of ≤ 130 m^2^ and each sampling location (N = 2936 for Sebastidae, 2654 for sponges, 2321 for corals) was ascribed to a 1-km^2^ planning unit within the standardized grid used to design the MPA network (N = 632 for Sebastidae, 525 for sponges, 529 for corals, 516 inclusive of surveys for all taxonomic groups).

**Table 1:** Survey methods used for data collection.

Survey method	Sampling years	Depth, m (mean)	Key characteristics	Data used in current analyses	Notes
Shallow diver transects^32,33^	2013, 2015–2021	5–35 (21)	Belt transects (30 m $\times$ 4 m $\times$ 4 m, or 480 m^3^), along depth contours	Relative density (count/480 m^3^) of fish and structural corals, by species Percent cover category of large-bodied sponges, aggregated for all Hexactinellidae and Demospongiae	Larger, older rockfishes^38^ and most structural corals^65^ tend to be deeper than the max. depth of dive surveys. Consistent with earlier publications^33^, analyses excluded fish < 10 cm-long. Sponge cover categories: 0 = 0%; 1 = 1- 25%; 2 = 26–50%; 3 = 51–75%; 4 = 76–100%
Mid-depth video transects^32,50,51^	2015–2018	15–200 (67)	Belt transects of variable size were divided into bins covering 75–130 m^2^ (mean = 116 m^2^) to reduce depth and habitat variability within spatial units. (Bins < 75 m^2^ are end cuts and bins > 130 m^2^ reflect GPS data gaps; analyses exclude both.) Parallel laser beams (10-cm apart) provide a distance scale	Relative density (count/m^2^) of fish and structural corals Percent cover category of large-bodied sponges, aggregated for all Hexactinellidae and Demospongiae (see dive transects for category values) Height of coral colony (distance from base to highest branch tip)	Fish counts were corrected for species detection biases (i.e., attraction to laser beams)^50,51^. Camera lacks panning/tilting ability and depth capacity of BOOTS camera (see below). The lower bound for bin size in earlier analyses^32^ was 100 m^2^, which we lowered to 75 m^2^ to not exclude some coral-rich areas. Heights for the coral *Calcigorgia* spp. were measured from 32 images in which the “flat” aspect of corals was perpendicular to the camera. (Other coral taxa were measured from Deep video)
Deep video transects (BOOTs)^35^	2018	100–500 (253)	Belt transects varied widely in area but were divided into similar size bins, as described for mid-depth video transects. Parallel laser beams (10-cm apart) provide a distance scale	Relative density (count/m^2^) for fish, structural corals, and large-bodied sponges Height of coral colony	Fish counts were corrected for species detection biases^50,51^. Coral heights were measured from 20 randomly selected images per taxon in which “flat” aspect of corals was perpendicular to the camera. Transect bins averaged 120 m^2^
Hook-and-line^34^	2006–2007; 2013–2015	15–205 (57)	Standardized gear fished the bottom for 15-min or 30-min sampling sessions	Relative density (count/min) for each fish species	During 2006–2007 data were collected by the Heiltsuk Nation prior to CCIRA’s inception

For full description of each method and its suite of data, see references in first column.

Although sampling encompassed 11 years (2006–2007, 2013–2021: Table 1), 84% of 1-km^2^ planning units were sampled during only one year (Appendix S2). Analyses, therefore, focus on spatial variability in species distributions and do not address temporal variability within planning units. When all years and methods are combined, 1-km^2^ planning units had a median of 3 samples (range = 1 to 80, *Q*_1_ = 2, *Q*_3_ = 6) (i.e., sum of dive transects, video sub-transects, and hook-and-line sessions). Supplementary Data Set 1 reports sampling effort by 1-km^2^ planning unit, survey type, and year (see Data Availability for link to these data).

For each 1-km^2^ planning unit, *u*, we calculated hotspot indices for Sebastidae (*B*_*SEB,u*_), structural corals (*B*_*Cor,u*_), and large-bodied sponges (*B*_*Sp,u*_). These indices did not consider cup corals, whip-like corals or encrusting corals or sponges.

As detailed below (Eqs. 1–4), each species of Sebastidae or genera of corals contributed to *B*_*SEB,u*_ or *B*_*Cor,u*_, according to their abundance weighted by *W*_*t*_: a conservation prioritization score based on taxon characteristics. For the 26 species of Sebastidae that we observed, *W*_*t*_ equaled the sum of scores for (1) fishery vulnerability, using intrinsic population growth rate, *r*, as a proxy variable^52,53^, (2) depletion level, using the ratio of recent biomass to unfished biomass as a proxy variable, (3) ecological role, with trophic level as proxy, and (4) evolutionary distinctiveness^14^ (Table 2; Appendix S3). Because several rockfishes are very long-lived (i.e., have low values for *r*) and depleted, maximum potential scores were twice as large for fishery vulnerability and depletion level than for ecological role and evolutionary distinctiveness. Data for depletion level and evolutionary distinctiveness were unavailable for some species, and score calculations (detailed in Table 2) account for missing values (Appendix S3).

**Table 2:** Criteria and equations used to calculate the conservation prioritization score, *W*_*t*,_ for each species of Sebastidae and for each taxa of structural corals.

Taxonomic group	Criteria	Proxy variable	Score
Sebastidae (rockfish and thornyheads)	Vulnerability	Intrinsic population growth rate, *r*	$Score_{1} = x_{1} \left( {\frac{\frac{1}{r}}{{max\left( \frac{1}{r} \right)}}} \right),$ where $x_{1} = 4$
	Depletion level	*B*_*y*_*/B*_*0*_ (median estimate of spawning biomass during year *y*, divided by the “unfished” biomass)	${Score}_{2}={x}_{2}\left(\frac{1-\left(\frac{{B}_{y}}{{B}_{0}}\right)}{max\left(1-\left(\frac{{B}_{y}}{{B}_{0}}\right)\right)}\right),$ where ${x}_{2}=4$
	Ecological role	Trophic Level, *TL*	${Score}_{3}={x}_{3}\left(\frac{TL-\mathrm{min}(TL)}{\mathrm{max}(TL)-\mathrm{min}(TL)}\right),$ where ${x}_{3}=2$. A min–max normalization is used to highlight differences between planktivores and upper-level predators within the relatively narrow range of TL values for Sebastidae
	Evolutionary distinctiveness, *ED*	None (direct measure^14^)	${Score}_{4}={x}_{4}\left(\frac{ED}{\mathrm{max}(ED)}\right),$ where ${x}_{4}=2$
	Overall species score, *W*_*t*_		${W}_{t}=\frac{\sum_{i=1}^{4}{Score}_{i}}{\sum_{i=1}^{4}{x}_{i}}$ Where ${x}_{i}=0$ if ${Score}_{i}$ cannot be calculated for that species (i.e., missing data for proxy variable)
Structural corals	Overall species score, *W*_*t*_	Mean height, *h* (cm)	${W}_{t}=\frac{h}{\mathrm{max}(h)}$

For the 6 genera of structural corals analyzed (Appendix S4), *W*_*t*_ depended on mean height (estimated from video transect images: Table 1), which correlates positively with vulnerability to physical damage from bottom-contact fishing gear (including longer time to recovery)^20,54,55^ and with strength of ecological role (e.g., amount of biogenic habitat and carbon sequestration increases with height)^44,56^ (Table 2, Appendix S4). *W*_*t*_ for corals did not include depletion level due to lack of data.

The hotspot index for large-bodied sponges, *B*_*Sp,u*_ did not differentiate between species characteristics (i.e., ${W}_{t}=1$) and we pooled the abundances of all observed species of Hexactinellidae (*Aphrocallistes vastus, Farrea occa, Heterochone calyx, Rhabdocalyptus dawsoni*, *Staurocalyptus dowlingi*) and Demospongiae (*Mycale* cf *loveni*). This approach is consistent with regional fishery bodies worldwide, which treat large-bodied sponges as a single functional group^57^.

To derive hotspot indices for each taxonomic group (Sebastidae, structural corals, or large-bodied sponges), we first developed a set of candidate generalized linear mixed models (GLMM) to explain relative abundance data for rockfish, corals, and sponges. For each GLMM, we estimated ${\lambda }_{t,i,l}$, the expected counts (or expected percent cover) for taxa *t* obtained with survey method *i* at point location *l*. (Point locations are individual dive transects, video transect bins, or hook-and-line timed sessions: Table 1.) Specifically,

1 $${\lambda }_{t,i,l}=g\left(\beta {X}_{t,i,l}\right)$$

2 $${C}_{t,i,l}\mathrm {\, or \,} {D}_{t,i,l}\sim f\left({\lambda }_{t,i,l}\right)$$

where *g* was the link function for the GLMM and *f* the distribution for the likelihood function modelling either the observed counts *C* (negative binomial) for Sebastidae and structural corals or a combination of counts (negative binomial) and percent cover *D* (beta distribution) for large-bodied sponges. We used multiple GLMMs to model large-bodied sponges because deep video transects recorded actual counts whereas dive or mid-depth video transects recorded percent cover categories (Table 1).

For each taxonomic group, we estimated a set of coefficients $\beta$ for the vector of *X* covariates that best estimated counts or percent cover. Our hypothesized covariates included the 1-km^2^ planning unit (modelled as a random intercept to control for repeated measures within a given planning unit), survey method, depth (including both linear or a 2^nd^ order polynomial), and taxa. Each GLMM controlled for sample effort as an offset—effort was measured either as area covered by dive transects or video bins, or the duration of hook-and-line sessions. We also tested for possible covariate’s effects on the dispersion parameter (for the negative binomial GLMMs) and zero-inflation terms (for both the negative binomial and beta GLMMs). The best set of covariates to predict counts or percent cover were then chosen based on AIC model selection criteria. All models were fitted using ‘glmmTMB’^58^ in R version 4.0.2^59^, and simulated residuals and diagnostic tests performed for each best-fit model using the package ‘DHARMa’^60^. For example, our best model for Sebastidae counts predicted 2% fewer zero counts than were observed.

We applied depth and survey method selectivity criteria to reduce excessive zeroes in the count data that may be biologically unjustified (Appendix S5). For all taxon, if *i* detected *t*, then the method was valid for that taxon. If *i* did not detect *t* and *t* is a Sebastidae, then the method was valid (i.e., count = 0) only if the overall 10th and 90th percentiles of depths sampled by that method encompassed the expected depth range of *t* (Appendix S5). If *i* did not detect *t* and *t* is a coral or sponge (which are rarer than Sebastidae), then the method is valid only if the depth of the sampling event exceeded or equaled the minimum expected depth of *t*. Also, hook-and-line gear cannot systematically sample sessile benthic organisms or planktivores and this method was valid only for non-planktivorous Sebastidae (Appendix S5).

Using the best-fit models from above, we calculated the expected count (or percent cover) per unit of effort, $\mu$, for taxa *t* observed with method *i* at each planning unit *u*:

3 $${\mu }_{t,i,u}=\frac{{\sum }_{l=1}^{{n}_{i,u}}\left({\lambda }_{t,i,l}\right)}{{\sum }_{l=1}^{{n}_{i,u}}\left({\mathrm{E}}_{t,i,l}\right)}$$

where ${n}_{i,u}$ was the total number of point locations sampled by that method within the planning unit and effort was either the cumulative area covered by dive or video surveys or the cumulative duration of hook-and-line sampling sessions within the planning unit. Because survey methods differed in their maximum values and potential biases (e.g., field of view is greater for divers than for video cameras; hook-and-line gear samples one fish at a time while visual methods can observe multiple fish simultaneously),${\mu }_{t,i,u}$ was rescaled as a min–max normalization,${\mu }_{t,i,u}^{^{\prime}}$ (i.e., difference between the observed value and the minimum value across all *u*, divided by the range of values across all *u*).

The hotspot index for each of Sebastidae, structural corals, and large-bodied sponges (denoted as taxonomic group *g*) was then calculated for each planning unit as:

4 $$B_{g,u}= \sum_{t=1}^{n_{s,g}}{\frac{\sum_{i=1}^{n_{m,g,u}}{\mu}_{t,i,u}'}{n_{m,g,u}}}W_t$$

where *W*_*t*_ was the taxon-specific weighing factor (Table 2, Appendices S3, S4), ${n}_{s,g}$ was the number of species in taxonomic group *g*, and ${n}_{m,g}$ was the number of valid methods to sample group *g*.

For each 1-km^2^ planning unit where all taxonomic groups were surveyed (N = 518), we then calculated the overall hotspot index:

5 $${B}_{o,u }=H{\sum }_{g=1}^{G}{B}_{g,u}.$$

where *H* is Shannon’s evenness index, with proportional abundance of each taxonomic group represented by *B*_*SEB,u*_, *B*_*Cor,u*_, and *B*_*Sp,u*_.

Hotspot index values were normalized as the proportion of the maximum value and converted to decile ranks. Relationships between decile ranks and index values were nonlinear (Appendix S6).

**Figure 2:**
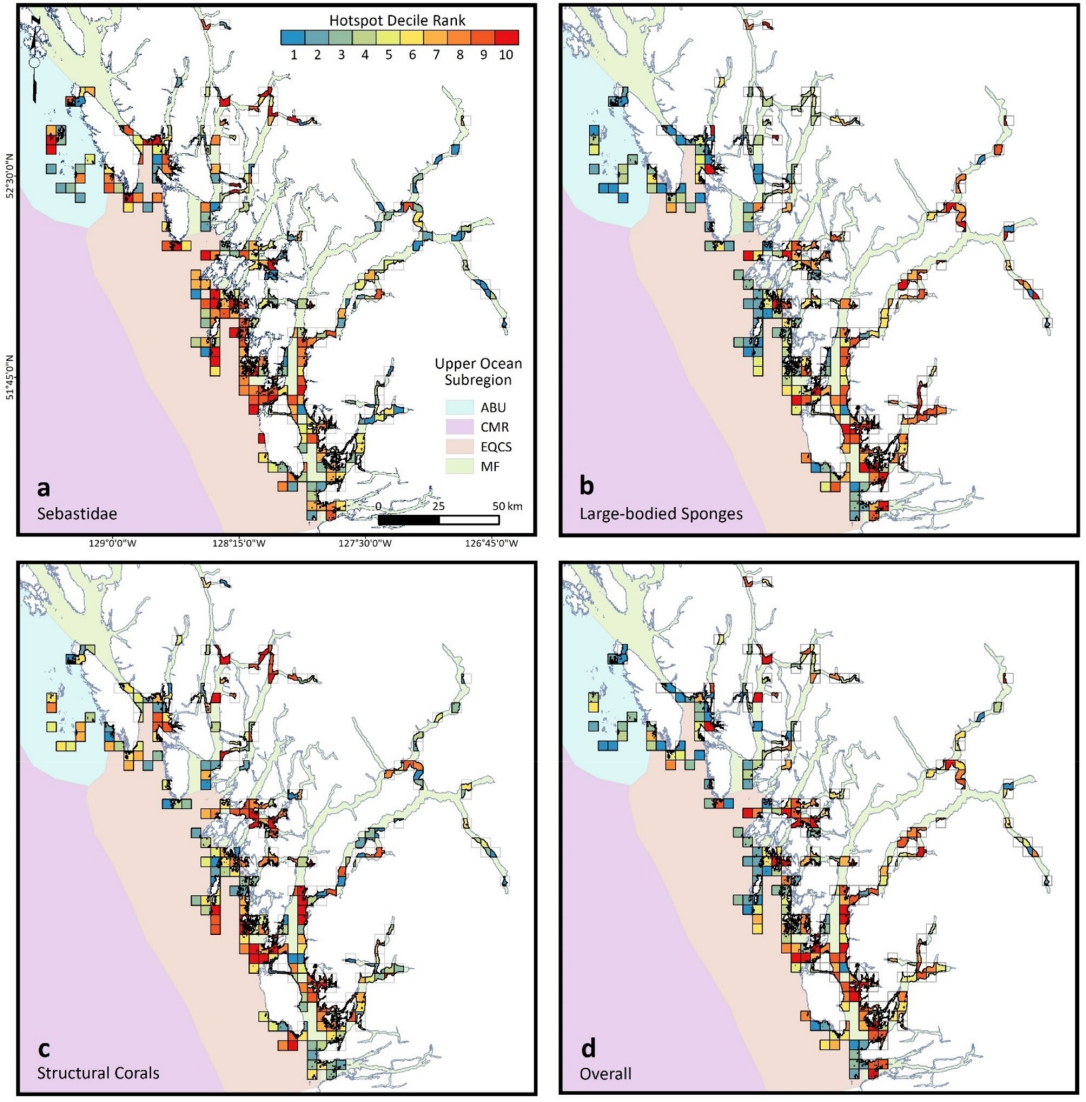
Spatial distribution of hotspot decile ranks within 16-km^2^ planning units (squares, except where faded over land), by species group (**a**) Sebastidae, (**b**) large-bodied sponges, (**c**) structural corals, and by Upper Ocean Subregions (*ABU* Aristazabal Banks Upwelling, *CSTM* Cape Scott Tidal Mixing, *EQCS* Eastern Queen Charlotte Sound, *MF* Mainland Fjords). Panel (**d**) displays decile ranks for the overall hotspot index, which integrates data from all taxonomic groups. Although primary analyses were conducted at the scale of 1-km^2^, First Nations of the Central Coast require this coarse scale for display of spatial data to protect sensitive locations. (Figure depicts outputs from Eqs. 4 and 5 and was created with ArcGIS Desktop, Version 10.8.1: https://www.esri.com/en-us/arcgis/products/arcgis-desktop/overview).

For Sebastidae, large-bodied sponges, and the overall hotspot index, we defined hotspots as planning units containing decile ranks 9 or 10: criterion which we deemed appropriate for the small spatial scales of conservation planning being used for the central portion of the Northern Shelf Bioregion (16-km^2^ planning units in Fig. 2). We are aware that other studies define hotspots based on a narrower range of values (e.g., top 10%^26^; top 2.5%^28^) but their context is generally one in which conservation planning is done at a much greater scale (e.g., ≈50,000-km^2^ grid cells^26^;1° latitude × 1° longitude grid cells^28^). For structural corals, which had near-zero index values in all but the top-ranking planning units (Appendix S6), we defined hotspots as planning units containing decile rank 10.

Maximum depths sampled within planning units were deepest in the Mainland Fjord and shallowest in the Aristazabal Banks Upwelling Upper Ocean Subregion (Appendix S7). Accordingly, we used multiple logistic regression implemented with the ‘*glm*’ function in R to estimate the probabilities hotspot occurrence within 1-km^2^ planning units in relation to maximum depth sampled (including a 2nd-order polynomial) and Upper Ocean Subregion. Competing models were compared with AIC model selection procedures.

Following the directive of Central Coast First Nations, decile rank distributions were mapped as 16-km^2^ planning units, *u*_*16*_ (N = 283 for Sebastidae, 264 for sponges, 263 for corals, 260 inclusive of surveys for all taxonomic groups), thereby protecting sensitive locations that would be revealed at smaller scales. To do so, we took the average between the maximum index value and the mean of the remainder of index values among the 1-km^2^ planning units, *u*, contained within each *u*_*16*_, and converted these values into decile ranks. This approach balances conservation prioritization among *u*_*16*_ that may have good average index values for multiple *u*, and *u*_*16*_ with a single high-ranking *u* among multiple low-scoring *u*. Relationships between decile ranks and hotspot index values also were nonlinear at this scale (Appendix S6). The same hotspot definitions developed for *u* apply to *u*_*16*_.

Eighty one percent of 16-km^2^ planning units were sampled during only one or two years (Appendix S2). When all years and methods are combined, 16-km^2^ planning units had a median of 6 samples (range = 1 to 110, *Q*_1_ = 3, *Q*_3_ = 13). Supplementary Data Set 2 reports sampling effort by 16-km^2^ planning unit, survey type, and year (see Data Availability for link to these data).

## Results

**Figure 3:**
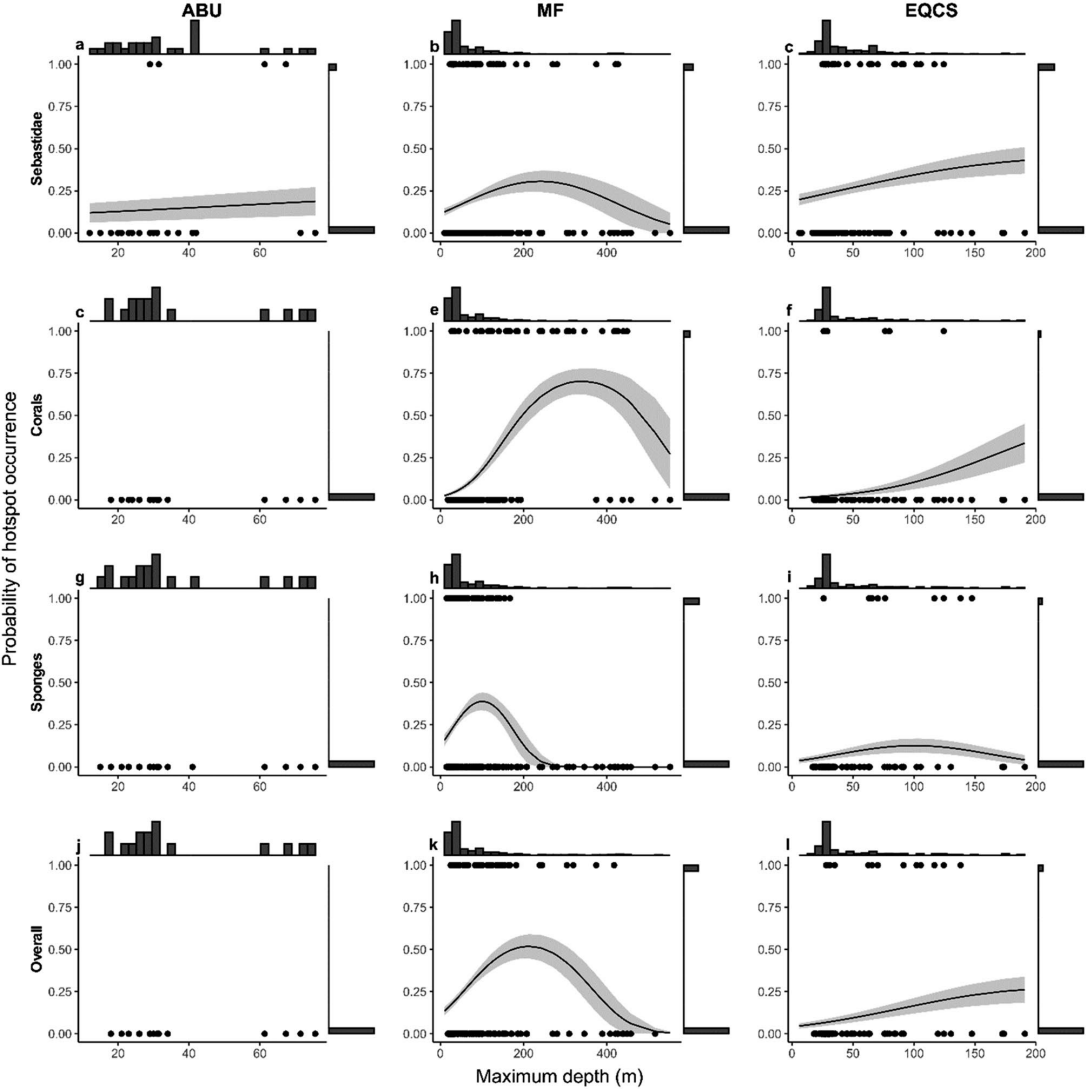
Probabilities of hotspot occurrence within 1-km^2^ planning units for (**a**–**c**) Sebastidae, (**d**–**f**) structural corals, (**g**–**i**) large-bodied sponges, and (**j**–**l**) overall, in relation to maximum depth sampled and Upper Ocean Subregion (*ABU* Aristazabal Banks Upwelling, *EQCS* Eastern Queen Charlotte Sound, *MF* Mainland Fjords). Circles are raw data (points overlap) and panel marginal histograms show their relative frequencies along each axis. Lines and shading are, respectively, logistic regression estimates with 95% confidence intervals (Table 3). Note that depth ranges differ between ocean subregions.

Field surveys recorded 101,145 individual Sebastidae, 8395 structural corals, 755 large-bodied sponges, and scored additional sponge clusters as percent cover categories (Appendix S8). For all species groups, hotspots spanned from oceanic areas to inland waters at the heads of fjords (Fig. 2) but were distributed unevenly across Upper Ocean Subregions and depths (Fig. 3; Appendix S9).

After accounting for depth, Sebastidae hotspots were more likely to occur at Eastern Queen Charlotte Sound than at other Upper Ocean Subregions (Table 3). At a depth of 60 m (which we sampled adequately throughout the study area: Appendix S7), probabilities of hotspot occurrence at Eastern Queen Charlotte Sound were 1.7 times and 1.6 times greater than at Aristazabal Banks Upwelling and Mainland Fjords, respectively (Fig. 3a–c; Table 3). Consistent with this result, for the 8 species in the top 25% of conservation prioritization scores (*W*_*t*_ ≥ 0.54: Appendix S3), expected counts, ${\lambda }_{t,i,l}$, were highest, on average, for 4 species at Eastern Queen Charlotte but only for 2 species at each of Mainland Fjords and Aristazabal Banks Upwelling (Appendix S10). However, three of these species—*S. borealis*, *S. aleutianus/melanostictus* and *S. babcocki*—have expected depths of 150 m or greater (Appendix S6); these depths were not sampled at Aristazabal Banks Upwelling (Appendix S7), which might have contributed to an underestimate of Sebastidae hotspots in that Upper Ocean Subregion.

**Table 3:** Logistic regression results examining probabilities of hotspot occurrence within 1-km^2^ planning units.

Response variable	Predictor	Estimate	SE	Odds Ratios (relative to MF)
Sebastidae hotspot	Maximum depth sampled	1.03E−02	3.83E−03	
	Maximum depth sampled (2nd-order)	−2.15E−05	9.91E−06	
	ABU	−7.38E−02	0.56	1.08
	EQCS	5.88E−01	2.17E−01	0.56
Coral hotspot	Maximum depth sampled	2.83E−02	4.75E−03	
	Maximum depth sampled (2nd-order)	−4.16E−05	1.01E−05	
	EQCS	6.14E−01	4.74E−01	1.85
Sponge hotspot	Maximum depth sampled	2.97E−02	1.03E−02	
	Maximum depth sampled (2nd-order)	−1.48E−04	5.75E−05	
	EQCS	−1.48	0.35	4.39
Overall hotspot	Maximum depth sampled	2.00E−02	4.27E−03	
	Maximum depth sampled (2nd-order)	−4.70E−05	1.19E−05	
	EQCS	−1.09	0.33	2.97

Effects of Upper Ocean Subregions (*ABU* Aristazabal Banks Upwelling, *EQCS* Eastern Queen Charlotte Sound), use Mainland Fjords (MF) as the reference. Models for corals, sponges, and overall hotspots did not include Aristazabal Banks Upwelling because no hotspots were recorded in these contexts (see Fig. 3).

Hotspots for structural corals and for large-bodied sponges were, after accounting for depth, more likely to occur at Mainland Fjords than at other Upper Ocean Subregions (Table 3). At a depth of 77 m (the 90th percentile for sampled depths at Eastern Queen Charlotte Sound: Appendix S7), probabilities of hotspot occurrence at Mainland Fjords for corals and sponges were, respectively, 1.8 times and 3.3 times greater than at Eastern Queen Charlotte Sound (Fig. 3; Table 3). Consistent with this result, the three coral taxa for which expected depths were adequately sampled at both Upper Ocean Subregions (Appendices S5, S7)—*Calcigorgia* spp., *Paragorgia pacifica*, and *Stylaster* spp.—had higher expected counts, on average, at Mainland Fjords that at Eastern Queen Charlotte Sound (Appendix S10). However, the remainder of coral taxa—including the top-ranking coral for conservation prioritization, *Primnoa pacifica*—have expected depths of 180 m or greater (Appendix S6); these depths were adequately sampled only at Mainland Fjords (Appendix S7); which likely biased results. Similarly, we did not record hotspots for structural corals or large-bodied sponges at Aristazabal Banks Upwelling (Fig. 3), which likely is a false negative as sampling effort was lowest and shallowest (Appendices S7, S9) at this most remote of the three Upper Ocean Subregions.

Overall hotspots—those with high evenness and summed index values for the three taxonomic groups—were more likely to occur, after accounting for depth, at Mainland Fjords than at other Upper Ocean Subregions (Fig. 3, Table 3). For instance, at a depth of 77 m, the probability of overall hotspot occurrence at Mainland Fjords was 2.4 times greater than at Eastern Queen Charlotte Sound (Fig. 3; Table 3). As detailed above, however, the deep expected depths of some Sebastidae and structural corals with high scores for conservation prioritization were sampled only at Mainland Fjords.

Of 102 1-km^2^ planning units containing overall hotspots, 33 included independent hotspots for more than one taxonomic group: 6 for Sebastidae and structural corals, 7 for large-bodied sponges and structural corals, 18 for Sebastidae and large-bodied sponges, and 2 for all three taxonomic groups. Similarly, of 52 16-km^2^ planning units containing overall hotspots, 30 included independent hotspots for more than one taxonomic group: 8 for Sebastidae and structural corals, 6 for large-bodied sponges and structural corals, 13 for Sebastidae and large-bodied sponges, and 3 for all taxonomic groups.

For all taxonomic groups and for overall hotspots, depth had a unimodal effect on the probabilities of hotspot occurrence within 1-km^2^ planning unit (Fig. 3, Table 3). These probabilities increased initially with depth up to a peak—239 m for Sebastidae, 340 m for structural corals, 100 m for large-bodied sponges, and 212 m for overall hotspots—before declining with further depth. The unimodal effect of depth, however, was evident for Sebastidae, structural corals, and overall hotspots only at Mainland Fjords, the only Upper Ocean Subregion where sampled depths exceeded 200 m (Fig. 3, Appendix S7).

## Discussion

**Figure 4:**
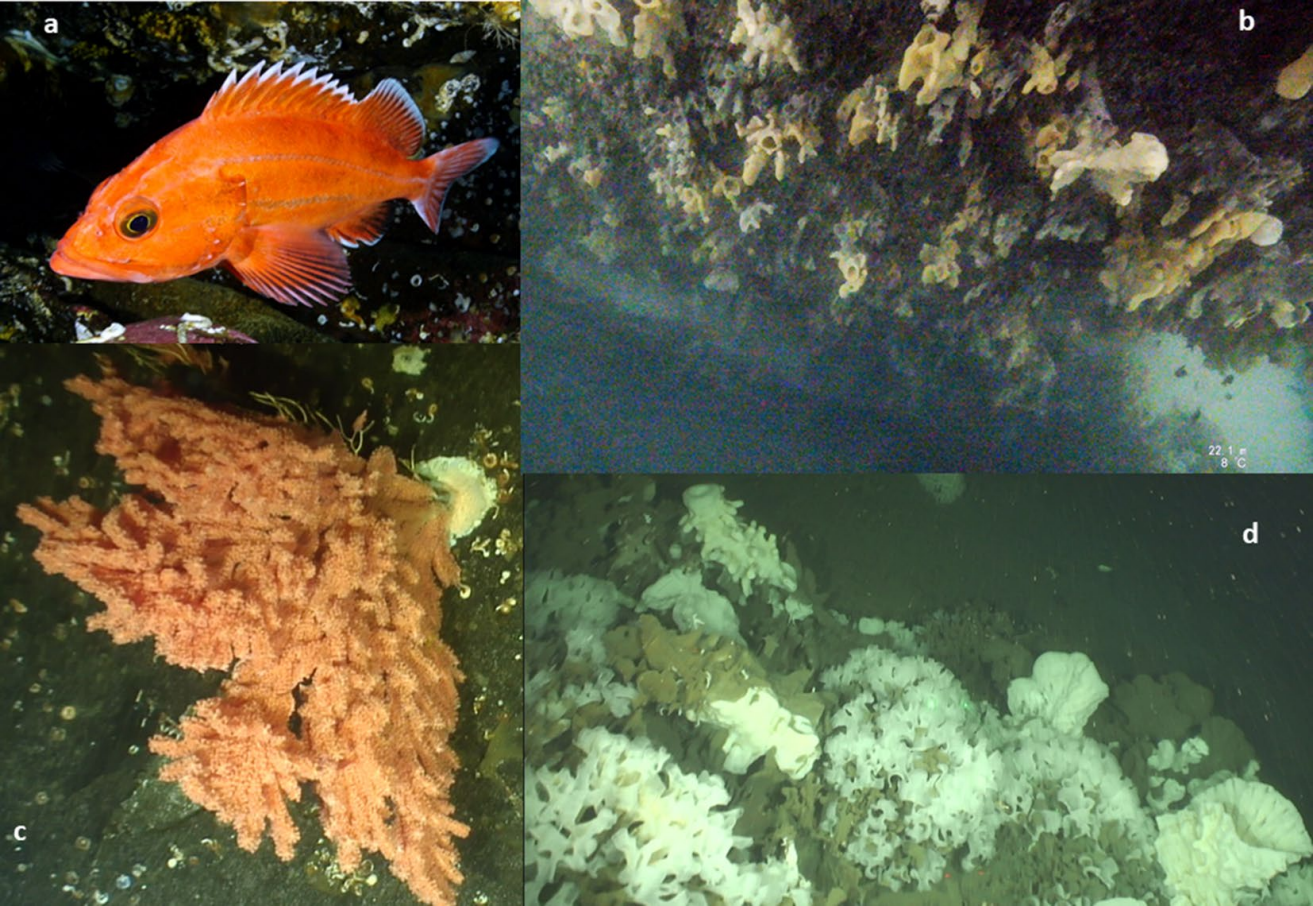
Examples from each taxonomic group observed during the study: (**a**) a Sebastidae, yelloweye rockfish (*Sebastes ruberrimus*); (**b**) large-bodied sponge garden on rocky wall ; (**c**) a structural coal, *Primnoa pacifica*; (**d**) large-bodied sponge bioherm reef. (All images obtained by the authors during data collection).

The pace of biodiversity loss is staggering^1^ and there is an urgent need to spatially protect biological hotspots^27^. Towards that end, our research highlights previously undocumented hotspot distributions for large-bodied sponges, structural corals, and long-lived fishes of the family Sebastidae (Fig. 4) along the central portion of Canada’s Northern Shelf Bioregion, particularly in the little-studied Mainland Fjords. The data are timely and are contributing to the design of the MPA network for the Northern Shelf Bioregion, which is nearing its final stages^22^. Given that commercial and recreational fisheries remain open throughout most of the bioregion, a well-designed MPA network could potentially mitigate fishery impacts on the species groups that we examined.

We recommend that 16-km^2^ planning units containing biological hotspots for any taxonomic group (i.e., decile ranks 9 or 10 for Sebastidae and large-bodied sponges; decile rank 10 for structural corals) be considered for the highest levels of spatial protection afforded by the MPA network (e.g., exclusion of commercial and recreational fisheries). If further conservation prioritization is required, then planning units containing overall hotspots—those with high evenness and summed index values for all taxonomic groups—should take precedence. Importantly, the notion of evenness is consistent with the worldview of many Indigenous peoples (including Central Coast First Nations) in which all species inherent to an ecosystem, not just those that provide direct sustenance, are valued^3^.

Because structural corals, large-bodied sponges, and long-lived species of Sebastidae are vulnerable to cumulative fishery impacts^18,19,61^ and the period between final network design and the enactment of MPA legislations can be protracted, prospective MPAs containing hotspots should be considered for interim protection, which DFO defines as “prohibiting any new human activities in the area for up to five years while scientific analysis and consultations continue^62^”. Additionally, planning units that did not meet hotspot criteria but that contain important biological values (e.g., decile ranks ≥ 6) should be considered for the siting of MPAs with lesser protection levels (e.g., some types of fisheries permitted).

Our results reflect the distribution of species that are ecologically important, fishery vulnerable (and depleted, in some cases), and/or evolutionary distinctive. The above recommendations, therefore, are consistent with the goals of the MPA network^48^ to protect upper-level predators and foundation species that influence community dynamics (Objective 1.1), to conserve “areas of high biological diversity (Objective 1.2),” and to aid the recovery of species with tenuous conservation status (Objective 1.5) (Appendix S1). Our recommendations also account for the distribution and relative abundance of small, planktivorous rockfishes (e.g., *S*. *emphaeus*, *S*. *jordani*) which, though weighted more lightly for conservation prioritization than upper level predators, also contributed to hotspot ranks. Our recommendations, however, reflect our scientific data, which are only one way of understanding the seascape. Our surveys, extensive as they are, encompassed only portions of spatial polygons ranked by First Nations as “critical” for the protection of cultural conservation priorities (Appendix S11), thereby failing to cover many locations known to local Indigenous peoples as biologically important. For that reason, it is paramount that Indigenous knowledge contributes substantially to the design of the MPA network^31^.

We also acknowledge that our analyses did not account for spatial variation in historical exploitation rates. It is plausible that some non-hotspot locations containing structurally complex rocky habitats, where many rockfish species are known to thrive^32,38^, are former hotspots that have been depleted but that could potentially be restored through spatial protection. The distribution of heterogenous, high quality habitats, therefore, should also inform site selection for the MPA network^32,37^, especially where such habitats do not overlap with current hotspots that are species-based.

The three Upper Ocean Subregions that we examined contain depths exceeding 200 m, and therefore encompass the expected depths of deeper-dwelling Sebastidae (e.g., *S. borealis*, *S. aleutianus/melanostictus* and *S. babcocki*) and structural corals (e.g., *Primnoa* spp) which scored high for conservation prioritization. Logistical constraints, however, allowed us to sample such depths only at Mainland Fjords. We caution that—although our data are the best available for conservation prioritization in the areas examined—future research that samples deep depths more uniformly across Upper Ocean Subregions will likely generate revised hotspots distributions.

The probabilities of hotspot occurrence were unimodal for all taxonomic groups. Whether these probabilities peaked at specific depths because of depth-dependent shifts in substrate or other factors requires further investigation. More generally, depth effects potentially reflect shifts in community composition or other community characteristics, as depth preferences differ between species of Sebastidae and between coral taxa (Appendix S5). In the case of large-bodied sponges, hotspots at shallow depths (≤ 35 m) occurred primarily on rocky walls at Mainland Fjords, where aggregations of *Aphrocallistes vastus* can be very dense (Fig. 4b), whereas at least some of the deeper hotspots likely consisted of bioherms (i.e., mound-shaped reefs where live sponges grow on the remains of dead sponges, creating a complex matric of habitats: Fig. 4d)^19,63^. Given the tremendous ecological importance of bioherms^19,45^, future research should delineate separate hotspot distributions for bioherms and sponge gardens (i.e., where live sponges grow on rocks).

The species groups that we examined are either sessile (sponges and corals) or include long-lived demersal fishes with strong site fidelity (many rockfishes^38^). Thus, they are likely to benefit from spatial protection, both directly (i.e., no fishery removals or impacts from bottom-contact fishing gear) and indirectly (increased resilience to ocean warming and other environmental shifts)^16^. Species important to the culture of Central Coast First Nations, however, span beyond those that we examined, and include migratory fishes^4,5^ that are more difficult to protect spatially (spawning aggregations excepted). The implication is that, alongside MPAs, improved fishery policies that extend beyond the narrow objectives of maximum sustained yield and that encompass broader ecosystem objectives also are needed to restore and protect biodiversity^64^.

## Supplementary Information

Supplementary Information.

## Data Availability

Computer code and data used in our analyses are available at https://doi.org/10.5281/zenodo.5555255.
