# Author Correction: Hotspots for rockfishes, structural corals, and large-bodied sponges along the central coast of Pacific Canada

**DOI:** 10.1038/s41598-022-05039-8

**Published:** 2022-01-11

**Authors:** Alejandro Frid, Madeleine McGreer, Kyle L. Wilson, Cherisse Du Preez, Tristan Blaine, Tammy Norgard

**Affiliations:** 1 Central Coast Indigenous Resource Alliance, Campbell River, BC Canada; 2 School of Environmental Studies, University of Victoria, Victoria, BC Canada; 3 Institute of Ocean Sciences, Fisheries and Oceans Canada, Sidney, BC Canada; 4 Pacific Biological Station, Fisheries and Oceans Canada, Nanaimo, BC Canada

Correction to: *Scientific Reports* 10.1038/s41598-021-00791-9, published online 09 November 2021

The original version of this Article contained an error in Equation 4, where

$${B}_{g,u}={\sum }_{t=1}^{{n}_{s,g}}{\sum }_{i=1}^{{n}_{m,g}}{\mu }_{t,i,u}^{\mathrm{^{\prime}}}{W}_{t}$$

now reads:

$${B}_{g,u }=\sum_{t=1}^{{n}_{s,g}}\frac{{\sum }_{i=1}^{{n}_{m,g,u}}{\mu }_{t,i,u}^{^{\prime}}}{{n}_{m,g,u}}{W}_{t}$$

The original Article has been corrected.

