# Supplementary material for: Hotspots for rockfishes, structural corals, and large-bodied sponges along the central coast of Pacific Canada
Source: Sci Rep. 2021 Nov 9;11:21944. doi: 10.1038/s41598-021-00791-9 (PMC8578610; doi:10.1038/s41598-021-00791-9)
Supplement: Supplementary file 1 — Supplementary Information. [file 41598_2021_791_MOESM1_ESM.pdf]

# Supplementary Material

## **Hotspots for rockfishes, structural corals, and large-bodied sponges along the central coast of Pacific Canada**

Alejandro Frid[1,2,*,§], Madeleine McGreer[1,§], Kyle L. Wilson[1,§], Cherisse Du Preez[3], Tristan Blaine[1], Tammy Norgard[4]

[1]Central Coast Indigenous Resource Alliance, Campbell River, BC, Canada
[2]School of Environmental Studies, University of Victoria, Victoria, BC
[3]Institute of Ocean Sciences, Fisheries and Oceans Canada, Sidney, BC, Canada
[4]Pacific Biological Station, Fisheries and Oceans Canada, Nanaimo, BC, Canada
[§]Equal contributions
*Corresponding Author: alejfrid@gmail.com

**Appendix S1**: Marine Protected Area Network Goals and Objectives for the Northern Shelf Bioregion. (Reproduced from Gale et al. 2019[1].)

| | |
|---|---|
| GOAL 1: TO PROTECT AND MAINTAIN MARINE BIODIVERSITY, ECOLOGICAL REPRESENTATION AND SPECIAL NATURAL FEATURES. | |
| 1.1. | Contribute to the conservation of the diversity of species, populations, and ecological communities, and their viability in changing environments. |
| 1.2. | Protect natural trophic structures and food webs, including populations of upper-level predators, key forage species, nutrient importing and exporting species, and structure-providing species. |
| 1.3. | Conserve areas of high biological diversity (species, habitat and genetic diversity). |
| 1.4. | Protect representative areas of every marine habitat in the bioregion. |
| 1.5. | Contribute to protection of rare, unique, threatened, and/or endangered species and their habitats. |
| 1.6. | Conserve ecologically significant areas associated with geological features and enduring/recurring oceanographic features. |
| 1.7. | Contribute to conservation of areas important for the life history of resident and migratory species. |
| GOAL 2: TO CONTRIBUTE TO THE CONSERVATION AND PROTECTION OF FISHERY RESOURCES AND THEIR HABITATS. | |
| 2.1. | Maintain or improve stock stability and productivity of species important for commercial, recreational, and Aboriginal fisheries. |
| 2.2. | Maintain within protected areas the natural size and age structure of fished populations. |
| 2.3. | Conserve habitat important to ensuring that the productive capacity and harvestable biomass of commercial, recreational, and Aboriginal fisheries species are maintained within healthy and resilient ecological limits. |
| GOAL 3: TO MAINTAIN AND FACILITATE OPPORTUNITIES FOR TOURISM AND RECREATION. | |
| 3.1. | Conserve sites compatible with, and of high value for, sustainable commercial tourism and recreation. |
| GOAL 4: TO CONTRIBUTE TO SOCIAL, COMMUNITY AND ECONOMIC CERTAINTY AND STABILITY. | |
| 4.1. | Enable economic development opportunities that are compatible with achievement of conservation objectives contained with Goal 1. |
| 4.2. | Maintain or enhance the long-term productivity, resilience and reliability of marine ecosystem goods and services. |
| 4.3. | Support opportunities for local communities to benefit from marine protected areas. |
| 4.4. | Strengthen participation and representation of communities and stakeholders in design, establishment and monitoring of the network. |
| 4.5. | Ensure that all marine protected areas have clearly defined objectives and effective management and monitoring measures. |
| 4.6. | Support effective MPA network governance, planning and management. |
| 4.7. | Establish modern and collaborative approaches to surveillance and compliance monitoring. |
| GOAL 5: TO CONSERVE AND PROTECT TRADITIONAL USE, CULTURAL HERITAGE AND ARCHAEOLOGICAL RESOURCES. | |
| 5.1. | Increase awareness and understanding of First Nations use and stewardship of resources and territories. |
| 5.2. | Represent marine areas of high cultural or historical value. |
| 5.3. | Contribute to conservation of species significant to First Nations and coastal communities including those important for cultural use and food security. |
| GOAL 6: TO PROVIDE OPPORTUNITIES FOR SCIENTIFIC RESEARCH, EDUCATION AND AWARENESS. | |
| 6.1. | Increase public awareness, understanding and stewardship of the marine environment. |
| 6.2. | Protect reference sites to support research and management. |
| 6.3. | Monitor and report on effectiveness of management actions across the network |

**Appendix S2.** Temporal distribution of sampling effort (all survey methods pooled). Data are for planning units where Sebastidae were sampled, which include the subsets of locations where corals and sponges were sampled.

| Years sampled | Number of planning units | |
|---|---|---|
| | 1-$km^2$ | 16-$km^2$ |
| 1 | 529 | 147 |
| 2 | 80 | 82 |
| 3 | 17 | 27 |
| 4 | 4 | 17 |
| 5 | 2 | 5 |
| 6 | 0 | 4 |
| 7 | 0 | 0 |
| 8 | 0 | 1 |

**Appendix S3**. Observed species of Sebastidae and their corresponding characteristics and conservation prioritization scores ($W_t$) used to calculate hotspot indices (see Table 2 in main text). Maximum known ages and lengths are as reported by Love et al. (2002)[2], except for the *Sebastes aleutianus/melanostictus* complex, which were sourced from DFO (2020)[3] and averaged between the two species. Trophic levels were obtained from FishBase[4] (averaged between species for *Sebastes aleutianus/melanostictus*). Evolutionary distinctiveness (ED) is as reported by Magnuson-Ford et al. (2009)[5]. Values for intrinsic population growth rate, *r*, were estimated from a generalized linear model[§§] based on maximum age and $L_\infty$ developed for all Sebastidae and based on global life history compendium insourced from Thorson (2020)[6]. Other data sources are indicated in the table. For $B_y/B_0$, parentheses indicate year of last assessment. See Table 2 (main text) for calculation of Scores1-4 and $W_t$. Species are ordered in decreasing order of $W_t$ values.

| Species | Max. age (yrs)[2] | Max length (cm)[2] | $L_\infty$, female (cm) | *r* | $B_y/B_0$ | Trophic level | ED | $Score_1$ | $Score_2$ | $Score_3$ | $Score_4$ | $W_t$ |
|---|---|---|---|---|---|---|---|---|---|---|---|---|
| *Sebastes ruberrimus* | 119 | 91 | 67.3[7] | 0.043 | 0.33[7] (2018) | 4.4 | 0.729 | 2.259 | 2.757 | 2.000 | 2.000 | 0.751 |
| *Sebastes borealis* | 157 | 120 | 67.0[8] | 0.029 | | 4.3 | 0.273 | 3.287 | | 1.833 | 0.749 | 0.734 |
| *Sebastes aleutianus/ melanostictus* | 165 | 75.45 | 53.0 | 0.024 | 0.29[3]* (2021) | 3.7 | 0.377 | 4.000 | 2.922 | 0.833 | 1.034 | 0.732 |
| *Sebastes babcocki* | 106 | 65.5 | 53.4[8] | 0.044 | 0.27[9] (2017) | 3.8 | 0.445 | 2.198 | 3.004 | 1.000 | 1.221 | 0.619 |
| *Sebastes paucispinis* | 50 | 98 | 81.3[8] | 0.152 | 0.028[10] (2020) | 3.5 | 0.698 | 0.635 | 4.000 | 0.500 | 1.915 | 0.587 |
| *Sebastes pinniger* | 84 | 76 | 59.6[8] | 0.064 | 0.25[11] (2008) | 3.8 | 0.483 | 1.513 | 3.086 | 1.000 | 1.325 | 0.577 |
| *Sebastes maliger* | 95 | 61 | 39.9[8] | 0.043 | 0.37[12] (2011) | 3.8 | 0.262 | 2.229 | 2.593 | 1.00 | 0.719 | 0.545 |
| *Sebastes nigrocinctus* | 116 | 61 | 47.4[§] | 0.036 | | 3.5 | 0.428 | 2.651 | | 0.500 | 1.174 | 0.541 |
| *Sebastes nebulosus* | 79 | 41 | 34.3[§] | 0.051 | | 3.9 | 0.347 | 1.892 | | 1.167 | 0.952 | 0.501 |
| *Sebastes flavidus* | 64 | 66 | 55.5[8] | 0.088 | 0.50[13] (2015) | 4.2 | 0.326 | 1.092 | 2.058 | 1.667 | 0.894 | 0.476 |
| *Sebastes elongatus* | 54 | 43 | 34.9[8] | 0.086 | | 3.7 | 0.673 | 1.123 | | 0.833 | 1.846 | 0.475 |

| | | | | | | | | | | | | |
|---|---|---|---|---|---|---|---|---|---|---|---|---|
| *Sebastes helvomaculatus* | 87 | 41 | 28.7[2] | 0.041 | | 3.7 | 0.213 | 2.376 | | 0.833 | 0.584 | 0.474 |
| *Sebastes melanops* | 50 | 60 | 49.0[14] | 0.115 | | 4.4 | 0.321 | 0.840 | | 2.000 | 0.881 | 0.465 |
| *Sebastes entomelas* | 60 | 59 | 54.8[8] | 0.096 | 0.37[15] (2019) | 3.7 | 0.373 | 1.008 | 2.593 | 0.833 | 1.023 | 0.455 |
| *Sebastes brevispinis* | 82 | 73 | 59.2[8] | 0.066 | 0.56[16] (2014) | 3.8 | 0.424 | 1.470 | 1.810 | 1.000 | 1.163 | 0.454 |
| *Sebastes miniatus* | 60 | 76 | 67.1[17] | 0.107 | | 3.9 | 0.452 | 0.902 | | 1.167 | 1.240 | 0.414 |
| *Sebastes proriger* | 55 | 51 | 37.9[18] | 0.088 | 0.62[18]* (2018) | 3.8 | 0.373 | 1.100 | 1.564 | 1.000 | 1.023 | 0.391 |
| *Sebastes caurinus* | 50 | 66 | 45.7[8] | 0.111 | | 4.1 | 0.274 | 0.873 | | 1.500 | 0.752 | 0.391 |
| *Sebastes auriculatus* | 34 | 56 | 44.2§ | 0.182 | | 4.0 | 0.337 | 0.530 | | 1.333 | 0.925 | 0.348 |
| *Sebastolobus alascanus* | 100 | 80 | 59.1§ | 0.050 | 0.79[19] (2016) | 3.6 | | 1.922 | 0.864 | 0.667 | | 0.345 |
| *Sebastes zacentrus* | 58 | 45 | 35.7[8] | 0.079 | | 3.7 | 0.240 | 1.221 | | 0.833 | 0.658 | 0.339 |
| *Sebastes diaconus* | 44 | 53 | 37.8[20] | 0.118 | | 3.7 | 0.373 | 0.816 | | 0.833 | 1.023 | 0.334 |
| *Sebastes jordani* | 32 | 35 | 28.1[2] | 0.154 | | 3.2 | 0.695 | 0.627 | | 0.000 | 1.907 | 0.317 |
| *Sebastes ciliatus/variabilis* | 67 | 53 | 42.2§ | 0.072 | | 3.4 | 0.197 | 1.351 | | 0.333 | 0.540 | 0.278 |
| *Sebastes wilsoni* | 26 | 23 | 21.6[8] | 0.176 | | 3.6 | 0.216 | 0.549 | | 0.667 | 0.593 | 0.226 |
| *Sebastes emphaeus* | 22 | 18.3 | 17.1[2] | 0.194 | | 3.3 | 0.221 | 0.499 | | 0.167 | 0.606 | 0.159 |

*Values are for southern stocks, which have the higher overlap with the study area[3].

§Data were unavailable for these species and values were estimated from the relationship between max. length (*L*) and female $L_\infty$ with data from the remainder of species: $L_\infty = e^{(0.51+0.81ln(L))}$, $R^2 = 0.87$.

§§$r$ was estimated as a function of maximum age (A) and female $L_\infty$ using data from all *Sebastidae* species in global analysis[6]:
$r = e^{(0.94-1.34ln(A)+0.55ln(L_\infty))}$, $R^2 = 0.91$.

**Appendix S4**. Structural corals analyzed and their corresponding heights (cm) and conservation prioritization scores ($W_t$). Height measurements are estimated from images made during towed video transects (see Table 1). Species are ordered in decreasing order of $W_t$ values.

| Order | Species | Mean ±SE height, cm (range) | $W_t$ |
|---|---|---|---|
| Alcyonacea | *Primnoa pacifica* | 42±6 (7-106) | 1.00 |
| Antipatharia | *Chrysopathes formosa* and *C. speciosa* | 21±3 (7-50) | 0.50 |
| Alcyonacea | **Calcigorgia* spp. | 10±0.5 (4-15) | 0.24 |
| Alcyonacea | *Paragorgia* spp. | 9±2 (1-44) | 0.21 |
| Alcyonacea | *Swiftia* cf *spauldingi* | 4±0 (2-6) | 0.10 |
| Anthoathecata | *Stylaster* spp. | 3±0 (2-5) | 0.07 |

*A specimen collected at a dive site was confirmed as *Calcigorgia spiculifera*.

**Appendix S5**. Expected depths and survey method validity for each taxonomic group. (Valid methods = 1, Not valid = 0, but see details in main text). Depth ranges for each survey method are the 10th and 90th percentiles of sampled depths. For Sebastidae, expected depths are the "typical" ranges described in Love *et al.* (2002)[2] or Butler *et al.* (2012)[21]. For structural corals, expected depths are from Stone & Cairns (2020)[22] unless indicated otherwise. For large-bodied sponges, minimum and maximum expected depths are, respectively, from Marliave (2015)[23] and Archer *et al.* (2020)[24]. ). Species are ordered as in Tables S3 and S4.

| Taxa | Expected depth (m) | | Survey method valid for species | | | |
|---|---|---|---|---|---|---|
| | Min. | Max | Hook & line (18-78 m) | Shallow diver transect (15-30 m) | Mid-depth video transect (25-119 m) | Deep video transect (126-435 m) |
| ***Sebastidae** | | | | | | |
| *Sebastes ruberrimus* | 91 | 180 | 1 | 1 | 1 | 1 |
| *Sebastes borealis* | 300 | 600 | 0 | 0 | 1 | 1 |
| *Sebastes aleutianus/ melanostictus* | 150 | 450 | 0 | 0 | 1 | 1 |
| *Sebastes babcocki* | 150 | 350 | 0 | 0 | 0 | 1 |
| *Sebastes paucispinis* | 95 | 225 | 0 | 1 | 1 | 1 |
| *Sebastes pinniger* | 80 | 200 | 1 | 1 | 1 | 1 |
| *Sebastes maliger* | 10 | 130 | 1 | 1 | 1 | 1 |
| *Sebastes nigrocinctus* | 30 | 298 | 1 | 1 | 1 | 1 |
| *Sebastes nebulosus* | 10 | 128 | 1 | 1 | 1 | 1 |
| *Sebastes flavidus* | 90 | 180 | 1 | 1 | 1 | 1 |
| *Sebastes elongatus* | 100 | 300 | 1 | 1 | 1 | 1 |
| *Sebastes helvomaculatus* | 80 | 350 | 0 | 0 | 1 | 1 |
| *Sebastes melanops* | 0 | 76 | 1 | 1 | 1 | 0 |
| *Sebastes entomelas* | 0 | 200 | 1 | 1 | 1 | 1 |
| *Sebastes brevispinis* | 100 | 300 | 1 | 1 | 1 | 1 |
| *Sebastes miniatus* | 50 | 150 | 1 | 1 | 1 | 1 |
| *Sebastes proriger* | 55 | 300 | 1 | 1 | 1 | 1 |

| | | | | | | |
|---|---|---|---|---|---|---|
| *Sebastes caurinus* | 0 | 70 | 1 | 1 | 1 | 0 |
| *Sebastes auriculatus* | 0 | 70 | 0 | 1 | 1 | 0 |
| *Sebastolobus alascanus* | 200 | 800 | 0 | 0 | 0 | 1 |
| *Sebastes zacentrus* | 200 | 300 | 0 | 0 | 1 | 1 |
| §*Sebastes diaconus* | 0 | 90 | 1 | 1 | 1 | 0 |
| §*Sebastes jordani* | 150 | 270 | 0 | 1 | 1 | 1 |
| *Sebastes ciliatus/variabilis* | 100 | 300 | 0 | 1 | 1 | 1 |
| *Sebastes wilsoni* | 60 | 150 | 1 | 1 | 1 | 1 |
| §*Sebastes emphaeus* | 10 | 470 | 0 | 1 | 1 | 1 |
| **Corals** | | | | | | |
| ***Primnoa* spp. | 183 | 755 | 0 | 0 | 0 | 1 |
| *Chrysopathes formosa/speciosa.* | 417 | 914 | 0 | 0 | 0 | 1 |
| ¥*Calcigorgia* spp. | 18 | 512 | 0 | 1 | 1 | 1 |
| ¥*Paragorgia* spp. | 38 | 2022 | 0 | 1 | 1 | 1 |
| *Swiftia* spp. | 210 | 2779 | 0 | 0 | 0 | 1 |
| *Stylaster* spp. | 20 | 2124 | 0 | 1 | 1 | 1 |
| **Sponges** | | | | | | |
| Hexactinellidae and Demospongiae aggregated | 15 | 230 | 0 | 1 | 1 | 1 |

*Juveniles of some rockfish species may have much shallower distributions than the expected depth of adults and may be detectable by dive surveys.
§Planktivorous Sebastidae (i.e., hook-and-line sampling not valid).
**Depths are for *Primnoa pacifica* var. *willeyi*, which are distributed from the Aleutian Islands to British Columbia. See Table 1of Cairns & Bayer (2005)[25].
¥Depths are for *Calcigorgia spiculifera* and for *Paragorgia pacifica*: the most likely species in the area. The minimum depth for the latter is sourced from Lamb & Hanby (2005)[26], as the much shallower minimum depths reported by Stone & Cairns (2020)[22] appear to not be applicable to the study area.

**Appendix S6.** Hotspot index values within each rank, by species group and the overall hotspot index (columns). Top and bottom rows are for 1-km$^2$ and 16-km$^2$ planning units, respectively.

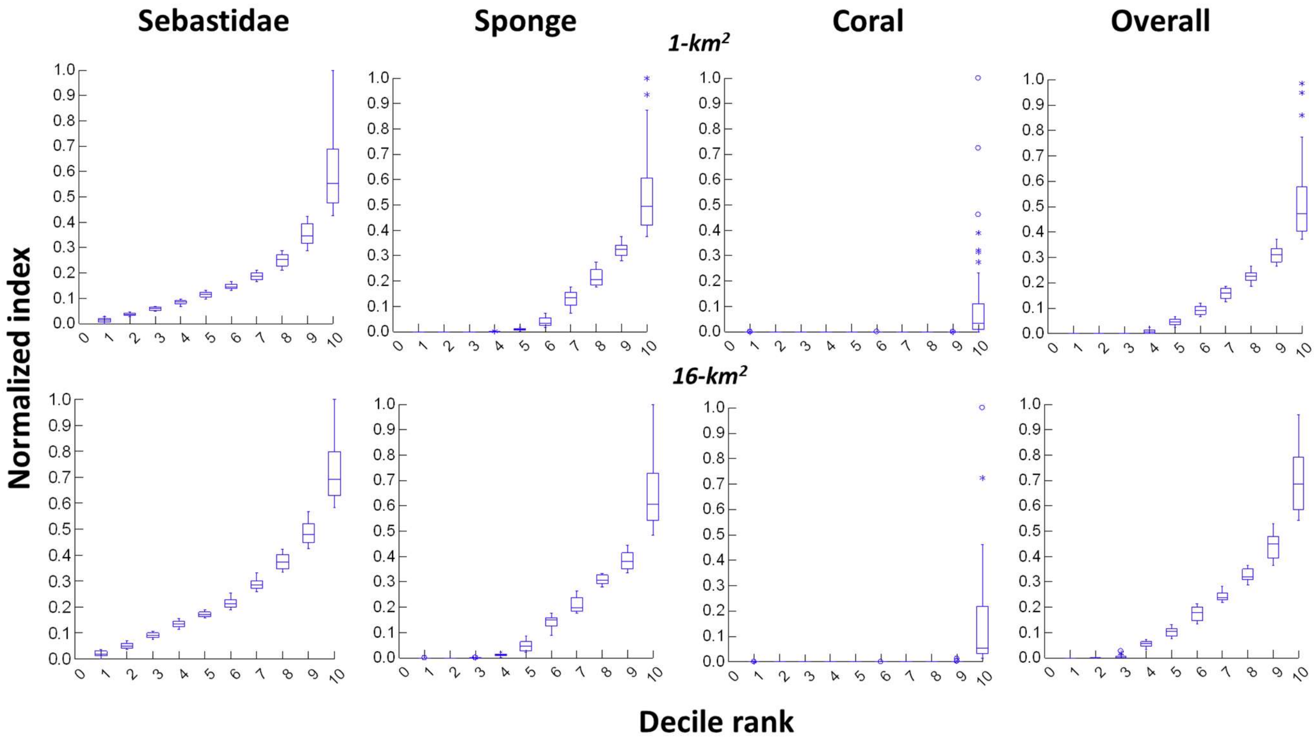

**Appendix S7**.

a) Maximum depths sampled within 16-km$^2$ planning units (squares, except where faded over land), overlayed with Upper Ocean Subregions[27] (ABU = Aristazabal Upwelling, CSTM = Cape Scott Tidal Mixing, EQCS = Eastern Queen Charlotte Sound, MF = Mainland Fjords). Panels are constrained to the survey methods (Table 1) applicable to each species group (left to right: Sebastidae, large-bodied sponges, structural corals).

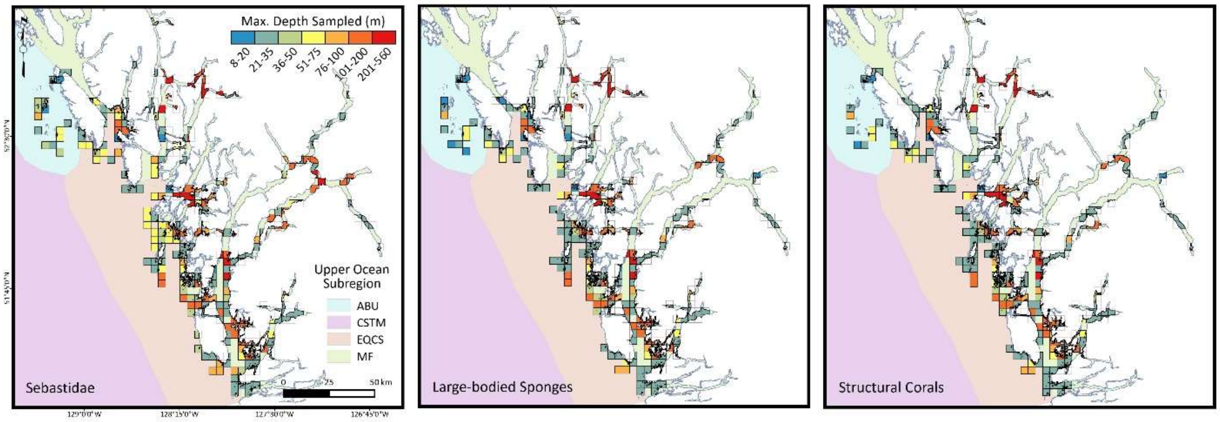


b) Maximum depth sampled within 1-km$^2$ planning units, by Upper Ocean Subregion and species group.

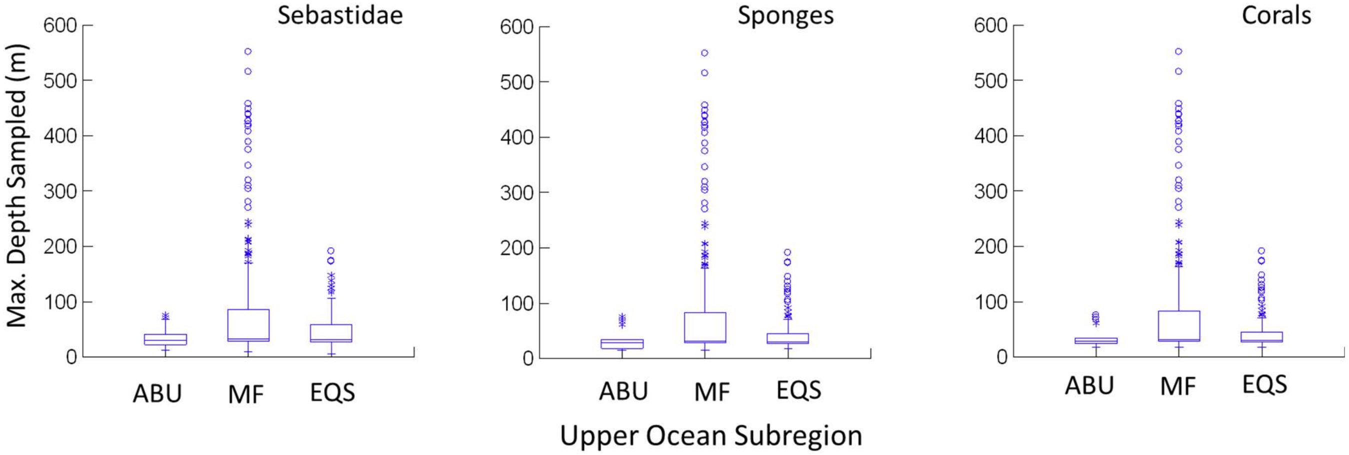

**Appendix S8.** Counts of individuals by taxon and survey method. Hook-and-line was not a valid method for corals and sponges (NA values). Sponge counts are reported only for deep video transects because other visual methods recorded percent cover categories. Species are ordered as in Tables S3 and S4.

| Taxon | Count | | | | |
|---|---|---|---|---|---|
| | Deep video transect | Shallow diver transect | Hook & line | Mid-depth video transect | Total |
| **Sebastidae** | | | | | |
| *Sebastes ruberrimus* | 10 | 420 | 80 | 128 | 638 |
| *Sebastes borealis* | 15 | 0 | 0 | 1 | 16 |
| *Sebastes aleutianus/ melanostictus* | 0 | 0 | 0 | 15 | 15 |
| *Sebastes babcocki* | 4 | 0 | 0 | 0 | 4 |
| *Sebastes paucispinis* | 0 | 915 | 0 | 8 | 923 |
| *Sebastes pinniger* | 0 | 731 | 10 | 99 | 840 |
| *Sebastes maliger* | 54 | 5949 | 168 | 886 | 7057 |
| *Sebastes nigrocinctus* | 2 | 265 | 4 | 27 | 298 |
| *Sebastes nebulosus* | 0 | 1666 | 17 | 59 | 1742 |
| *Sebastes flavidus* | 0 | 33978 | 22 | 1714 | 35714 |
| *Sebastes elongatus* | 34 | 2 | 0 | 46 | 82 |
| *Sebastes helvomaculatus* | 23 | 0 | 0 | 2 | 25 |
| *Sebastes melanops* | 0 | 9555 | 14 | 179 | 9748 |
| *Sebastes entomelas* | 0 | 30588 | 0 | 817 | 31405 |
| *Sebastes brevispinis* | 0 | 652 | 15 | 18 | 685 |
| *Sebastes miniatus* | 0 | 327 | 3 | 5 | 335 |
| *Sebastes proriger* | 90 | 911 | 2 | 250 | 1253 |
| *Sebastes caurinus* | 0 | 1724 | 99 | 38 | 1861 |
| *Sebastes auriculatus* | 0 | 7 | 0 | 4 | 11 |
| *Sebastolobus alascanus* | 3 | 0 | 0 | 0 | 3 |
| *Sebastes zacentrus* | 81 | 0 | 0 | 75 | 156 |
| §*Sebastes diaconus* | 0 | 1436 | 0 | 90 | 1526 |
| *Sebastes jordani* | 0 | 138 | 0 | 1 | 139 |
| *Sebastes ciliatus/variabilis* | 0 | 1375 | 0 | 10 | 1385 |
| *Sebastes wilsoni* | 0 | 24 | 0 | 44 | 68 |
| §*Sebastes emphaeus* | 0 | 4636 | 0 | 580 | 5216 |
| Total Sebastidae | 316 | 95299 | 434 | 5096 | 101145 |

| | | | | | |
|---|---|---|---|---|---|
| **Corals** | | | | | |
| *Primnoa pacifica* | 551 | 1 | NA | 0 | 552 |
| *Chrysopathes formosa/speciosa* | 26 | 0 | NA | 0 | 26 |
| *Calcigorgia* spp. | 0 | 181 | NA | 1277 | 1458 |
| *Paragorgia* spp. | 311 | 0 | NA | 110 | 421 |
| *Swiftia* cf *spauldingi* | 278 | 0 | NA | 0 | 278 |
| *Stylaster* spp. | 180 | 3818 | NA | 1662 | 5660 |
| Total corals | 1346 | 4000 | NA | 3049 | 8395 |
| **Large-bodied sponges** | 755 | % cover recorded | NA | % cover recorded | |

**Appendix S9.** Proportions of hotspot decile ranks by Upper Ocean Subregions, UOS (ABU = Aristazabal Upwelling, EQCS = Eastern Queen Charlotte Sound, MF = Mainland Fjords) and species group. Sample sizes indicate the number of 1-$km^2$ planning units sampled.

| Species group | UOS | Hotspot Index Rank | | | | | | | | | |
|---|---|---|---|---|---|---|---|---|---|---|---|
| | | 1 | 2 | 3 | 4 | 5 | 6 | 7 | 8 | 9 | 10 |
| Sebastidae | ABU (N = 28) | 0.07 | 0.21 | 0.18 | 0.18 | 0.04 | 0.04 | 0.00 | 0.14 | 0.04 | 0.11 |
| | MF (N = 415) | 0.12 | 0.10 | 0.10 | 0.11 | 0.12 | 0.10 | 0.10 | 0.08 | 0.09 | 0.08 |
| | EQCS (N = 189) | 0.06 | 0.08 | 0.09 | 0.07 | 0.07 | 0.11 | 0.12 | 0.14 | 0.12 | 0.14 |
| Large-bodied sponges | ABU (N = 18) | 0.12 | 0.08 | 0.17 | 0.23 | 0.29 | 0.12 | 0.00 | 0.00 | 0.00 | 0.00 |
| | MF (N = 375) | 0.01 | 0.03 | 0.03 | 0.05 | 0.09 | 0.09 | 0.13 | 0.17 | 0.20 | 0.19 |
| | EQCS (N = 132) | 0.03 | 0.06 | 0.13 | 0.16 | 0.10 | 0.17 | 0.14 | 0.04 | 0.02 | 0.16 |
| Structural corals | ABU (N = 17) | 0.12 | 0.12 | 0.06 | 0.12 | 0.00 | 0.29 | 0.18 | 0.12 | 0.00 | 0.00 |
| | MF (N = 374) | 0.10 | 0.09 | 0.10 | 0.11 | 0.10 | 0.07 | 0.08 | 0.11 | 0.11 | 0.12 |
| | EQCS (N = 138) | 0.10 | 0.13 | 0.10 | 0.07 | 0.12 | 0.16 | 0.14 | 0.07 | 0.07 | 0.04 |
| Overall | ABU (N = 16) | 0.25 | 0.19 | 0.13 | 0.25 | 0.13 | 0.06 | 0.00 | 0.00 | 0.00 | 0.00 |
| | MF (N = 370) | 0.08 | 0.08 | 0.06 | 0.09 | 0.10 | 0.10 | 0.11 | 0.13 | 0.12 | 0.12 |
| | EQCS (N = 130) | 0.15 | 0.14 | 0.21 | 0.12 | 0.09 | 0.11 | 0.07 | 0.03 | 0.05 | 0.05 |

**Appendix S10.** Average expected counts, $\lambda_{t,i,l}$, in each Upper Ocean Subregion (ABU = Aristazabal Banks Upwelling, EQCS = Eastern Queen Charlotte Sound, MF = Mainland Fjords) for (a) the 8 Sebastidae species with the top 25% of conservation prioritization scores ($W_t$≥0.54: Appendix S3), and (b) for structural corals. For each taxon, counts are normalized across Upper Ocean Subregions. In both panels, taxa are arranged from left to right in descending order of their $W_t$ values (see Appendix S3).

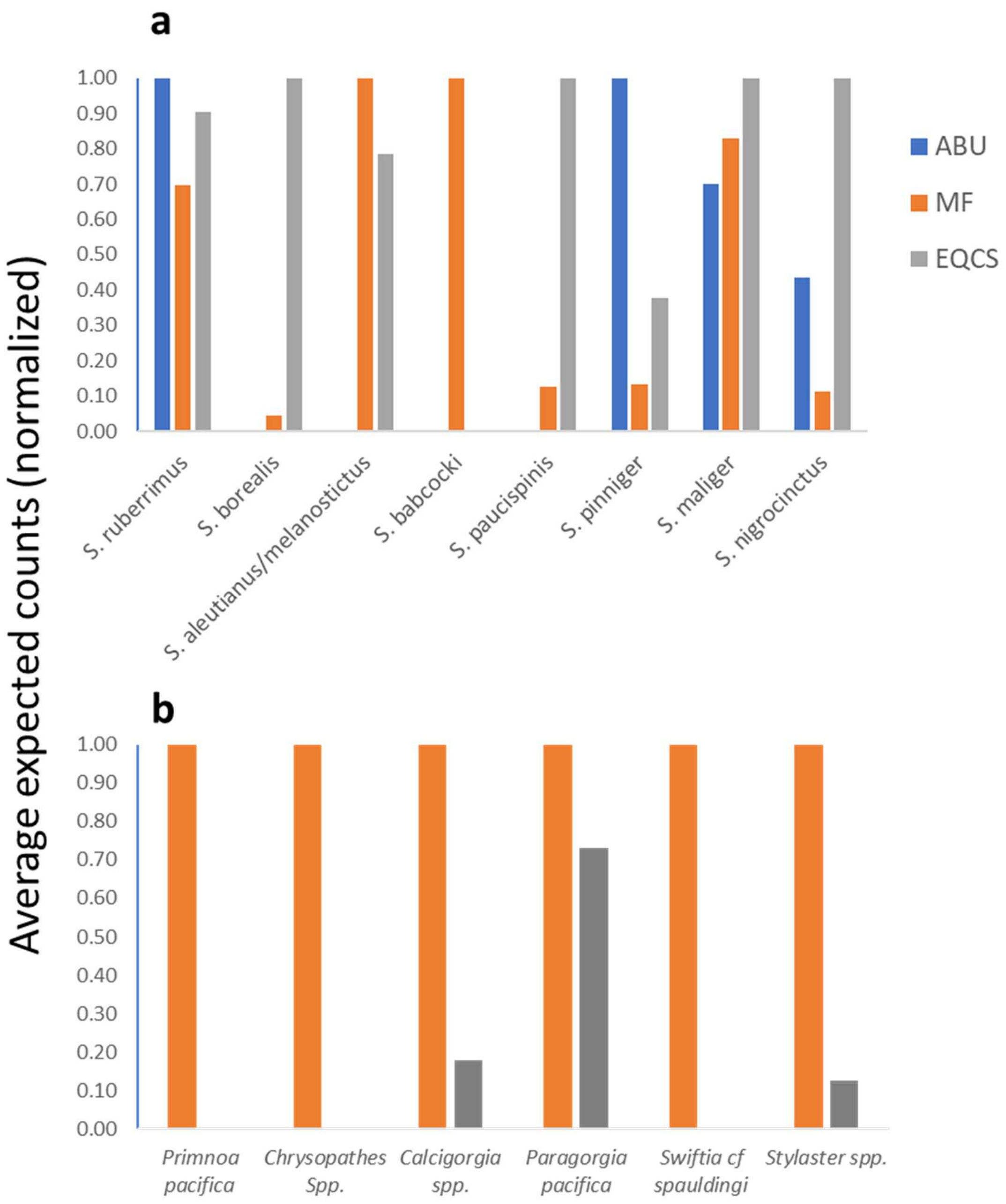

**Appendix S11**. Central Coast First Nations Cultural Conservation Priorities, as depicted in the SeaSketch Portal for Northern Shelf Bioregion MPA Network (last accessed 28-Sept-2021). (SeaSketch is a marine spatial planning tool designed for collaborative planning.). As stated in the metadata:

> This map layer is produced solely for the purposes of the marine planning and the Heiltsuk, Kitasoo/Xai'Xais, Nuxalk and Wuikinuxv Nations have not verified that all the facts and/or opinions expressed on this map are accurate. The Heiltsuk, Kitasoo/Xai'Xais, Nuxalk and Wuikinuxv Nations assert aboriginal title and rights, including ownership, jurisdiction and management over the lands, waters and resources, including the marine spaces, throughout our respective territories. Our indigenous laws and traditions hold our people responsible for ensuring that among other things, healthy sustainable territories, including the natural and cultural heritage, are passed on to future generations. All areas within our territories are culturally important to the Heiltsuk, Kitasoo/Xai'Xais, Nuxalk and Wuikinuxv Nations - any areas not listed in the Cultural Conservation Priority data layer have been rated as a moderate cultural conservation priority. The mapping of cultural values and cultural importance is a work in progress and is not definitive and is provided without prejudice.

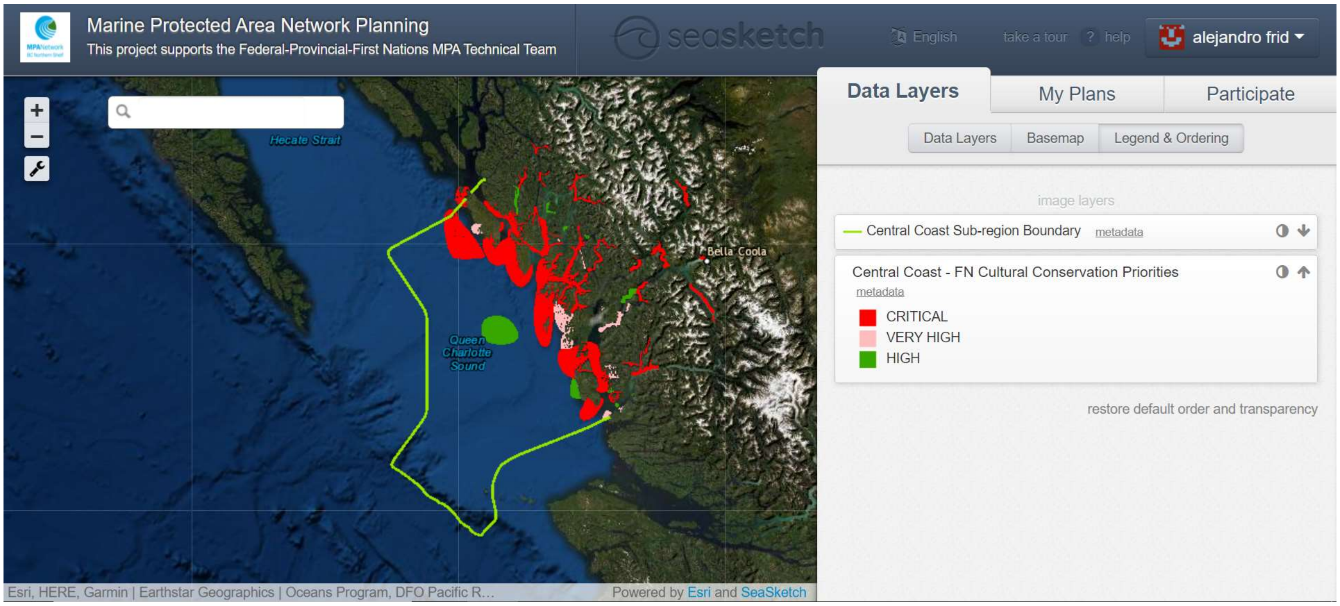

**Appendix S12**: References for supplementary material

1. Gale, K. S. P. *et al.* A framework for identification of ecological conservation priorities for MarineProtected Area network design and its application in the Northern Shelf Bioregion. *DFOCan. Sci. Advis. Sec. Res. Doc.* **2018/055**, 1–186 (2019).
2. Love, M., Yoklavich, M. & Thorsteinson, L. *The Rockfishes of the Northeast Pacific*. (University of California Press, 2002).
3. DFO. Rougheye/Blackspotted Rockfish (Sebastes aleutianus/melanostictus) Stock Assessment for British Columbia in 2020. *DFO Can. Sci. Advis. Sec. Sci. Advis. Rep.* **2020/047**, (2020).
4. Froese, R. & Pauly, D. FishBase. *World Wide Web electronic publication.* www.fishbase.org (2020).
5. Magnuson-Ford, K., Ingram, T., Redding, D. W. & Mooers, A. Ø. Rockfish (Sebastes) that are evolutionarily isolated are also large, morphologically distinctive and vulnerable to overfishing. *Biol. Conserv.* **142**, 1787–1796 (2009).
6. Thorson, J. T. Predicting recruitment density dependence and intrinsic growth rate for all fishes worldwide using a data-integrated life-history model. *Fish Fish.* **21**, 237–251 (2020).
7. Cox, S. P., Doherty, B., Benson, A. J., Johnson, S. D. N. & Haggarty, D. R. Evaluation of potential rebuilding strategies for Outside Yelloweye Rockfish in British Columbia. *DFO Can. Sci. Advis. Sec. Res. Doc.* **2020/069**, 135 (2020).
8. Anderson, S., EA, K. & Edwards, A. A reproducible data synopsis for over 100 species of British Columbia groundfish. *DFO Can. Sci. Advis. Secr. Res. Doc.* **2019/041**, 1–330 (2019).
9. Edwards, A. M., Haigh, R. & Starr, P. J. Redbanded Rockfish (Sebastes babcocki) stock assessment for the Pacific coast of Canada in 2014. . . *DFO Can. Sci. Advis. Sec. Res. Doc.* **2017/058**, v + 182 p (2017).
10. DFO. Bocaccio (Sebastes paucispinis) stock assessment for British Columbia in 2019, including guidance for rebuilding plans. *Can. Sci. Advis. Sec. Sci. Advis. Rep* **2020/025**, (2020).
11. Stanley, R. D., Starr, P. & Olsen, N. Stock assessment for Canary rockfish (Sebastes pinniger) in British Columbia waters. *Can. Sci. Advis. Sec. Res. Doc.* **2009/013**, (2009).
12. DFO. Stock Assessment And Recovery Potential Assessment For Quillback Rockfish (Sebastes Maliger) Along The Pacific Coast Of Canada. *DFO Can. Sci. Advis. Sec. Sci. Advis. Rep.* **2011/072**, (2012).
13. DFO. Yellowtail rockfish (Sebastes flavidus) stock assessment for the coast of British Columbia, Canada. *DFO Can. Sci. Advis. Sec. Sci. Advis. Rep.* **2015/010.**, (2015).
14. Cope, J. M. *et al. Assessments of California, Oregon and Washington Stocks of Black Rockfish (Sebastes melanops) in 2015*. (2016).
15. DFO. Widow Rockfish (Sebastes entomelas) stock assessment for British Columbia in 2019. *DFO Can. Sci. Advis. Sec. Sci. Advis. Rep.* **2019/044**, (2019).

16. Starr, P., Haigh, R. & Grandin, C. Stock assessment for Silvergray Rockfish (Sebastes brevispinis) along the Pacific coast of Canada. *Can. Sci. Advis. Secr. Res. Doc.* **2016/042**, (2016).

17. Hannah, R. W. & Kautzi, L. *Age, growth and female maturity of vermilion rockfish (Sebastes miniatus) from Oregon waters*. (2012).

18. Starr, P. J. & Haigh, R. Redstripe Rockfish (Sebastes proriger) stock assessment for British Columbia in 2018. *DFO Can. Sci. Advis. Sec. Res. Doc.* **2021/014**, 1–340 (2021).

19. Starr, P. J. & Haigh, R. Stock assessment of the coastwide population of Shortspine Thornyhead (Sebastolobus alascanus) in 2015 off the British Columbia coast. *DFO Can. Sci. Advis. Sec. Res. Doc.* (2017).

20. Dick, E. J. *et al. 2017. The Combined Status of Blue and Deacon Rockfishes in U.S. Waters off California and Oregon in 2017*. *Pacific Fishery Management Council* (2017).

21. Butler, J. L., Love, M. S. & Laidig, T. E. *A Guide to the Rockfishes, Thornyheads, and Scorpionfishes of the Northeast Pacific*. (University of California Press, 2012).

22. Stone, R. & Cairns, S. *Deep-Sea Coral Taxa in the Alaska Region: Depth and Geographical Distribution*. https://deepseacoraldata.noaa.gov/library/2020-regional-deep-sea-coral-species-list (2020).

23. Marliave, J. Cloud Sponge, Aphrocallistes vastus (Porifera: Hexactinellida), Fragment Healing and Reattachment. *Can. F. Nat.* **129**, 399–402 (2015).

24. Archer, S. K. *et al.* Foundation Species Abundance Influences Food Web Topology on Glass Sponge Reefs . *Frontiers in Marine Science* vol. 7 799 (2020).

25. Cairns, S. & Bayer, F. A review of the genus Primnoa (Octocorallia: Gorgonacea: Primnoidae), with the description of two new species. *Bull. Mar. Sci.* **77**, 225–256 (2005).

26. Lamb, A. & Hanby, B. *Marine Life of the Pacific Northwestle*. (Harbour Publishing, 2005).

27. Robinson, C. & McBlane, L. A summary of major upper ocean sub regions found within Parks Canada's five Natural Marine Regions on the Pacific coast of Canada. *BCMCA* (2013).
